# Impaired autophagic flux is associated with the severity of trauma and the role of A_2A_R in brain cells after traumatic brain injury

**DOI:** 10.1038/s41419-018-0316-4

**Published:** 2018-02-14

**Authors:** Xu-Jia Zeng, Ping Li, Ya-Lei Ning, Yan Zhao, Yan Peng, Nan Yang, Zi-Ai Zhao, Jiang-Fan Chen, Yuan-Guo Zhou

**Affiliations:** 1 Molecular Biology Center; State Key Laboratory of Trauma, Burn, and Combined Injury; Research Institute of Surgery and Daping Hospital, The Third Military Medical University, 10 Changjiang Zhilu, Chongqing, 400042 China; 2 Department of Neurology and Pharmacology, Boston University School of Medicine, Boston, MA 02118 USA

## Abstract

Recent studies have shown that after traumatic brain injury (TBI), the number of autophagosomes is markedly increased in brain cells surrounding the wound; however, whether autophagy is enhanced or suppressed by TBI remains controversial. In our study, we used a controlled cortical impact system to establish models of mild, moderate and severe TBI. In the mild TBI model, the levels of autophagy-related protein 6 (Beclin1) and autophagy-related protein 12 (ATG12)-autophagy-related protein 5 (ATG5) conjugates were increased, indicating the enhanced initiation of autophagy. Furthermore, the level of the autophagic substrate sequestosome 1 (SQSTM1) was decreased in the ipsilateral cortex. This result, together with the results observed in tandem mRFP-GFP-LC3 adeno-associated virus (AAV)-infected mice, indicates that autophagosome clearance was also increased after mild TBI. Conversely, following moderate and severe TBI, there was no change in the initiation of autophagy, and autophagosome accumulation was observed. Next, we used chloroquine (CQ) to artificially impair autophagic flux in the injured cortex of the mild TBI model and found that the severity of trauma was obviously exacerbated. In addition, autophagic flux and trauma severity were significantly improved in adenosine A_2A_ receptor (A_2A_R) knockout (KO) mice subjected to moderate TBI. Thus, A_2A_R may be involved in regulating the impairment of autophagic flux in response to brain injury. Our findings suggest that whether autophagy is increased after TBI is associated with whether autophagic flux is impaired, and the impairment of autophagic flux exacerbates the severity of trauma. Furthermore, A_2A_R may be a target for alleviating the impairment in autophagic flux after TBI.

## Introduction

Autophagy is a continuous process that proceeds from initiation to the digestion of the autophagosome^1, 2^. Hence, the autophagy process is also called autophagic flux, and the strength of the end result of this process is often viewed as representing the strength of autophagy. Although some researchers have correlated pathologically increased autophagy in some conditions with cell death, autophagy is regarded as a cytoprotective process under most conditions. Many nerve injuries, including brain trauma and injuries caused by neurodegenerative diseases, such as Parkinson’s disease and Alzheimer’s disease, are associated with reduced autophagy^3–6^.

Traumatic brain injury (TBI) is one of the most common types of brain injury. Many recent studies have shown that there is a marked increase in the number of autophagosomes in brain cells surrounding the wound following TBI. However, whether autophagy is enhanced or weakened by TBI remains controversial^7–15^. The results of some studies have shown that, in moderate TBI, the initiation of autophagy is unchanged, and autophagosomes accumulate in cells but do not exert a cytoprotective effect^16^. A separate report found that in mild TBI, the number of autophagosomes was increased and autophagy appeared to reduce brain damage^17^. We evaluated these two viewpoints and found that impaired autophagic flux might not occur in mild TBI, in which autophagosomes were effectively transformed into autolysosomes, ultimately enhancing autophagy. This finding suggests that the strength of autophagy is represented not only by the initiation of autophagy but also by normal autophagic flux. Our results, in combination with the results from numerous previous studies on autophagy after TBI^7–17^, demonstrate that the critical point at which autophagy becomes impaired after TBI is likely to lie between mild and moderate injury. TBI results in the production of a large amount of adenosine. Adenosine subsequently activates adenosine A_2A_ receptors (A_2A_Rs), thereby promoting an inflammatory reaction and brain edema, both of which are associated with the severity of trauma. Conversely, in moderate and severe TBI, A_2A_R inhibition exerts protective effects on both acute injury and chronic cognitive impairment^18^. Whether the role of A_2A_R in moderate TBI is related to the observed impairment of autophagic flux in brain cells remains unclear.

To explore the above questions, we first analyzed the levels of autophagy-related proteins and expression of autophagy-related genes to determine whether there are differences in autophagic flux among subjects with mild, moderate and severe TBI. Next, we compared the levels of autophagy-related proteins and expression of autophagy-related genes in brain tissues obtained from wild type (WT) and A_2A_R knockout (KO) mice after moderate TBI, and we found that A_2A_R contributes to the impairment of autophagic flux observed in brain cells in TBI.

## Results

### Different severities of trauma induce different levels of autophagic flux in the ipsilateral cortex

**Fig. 1:**
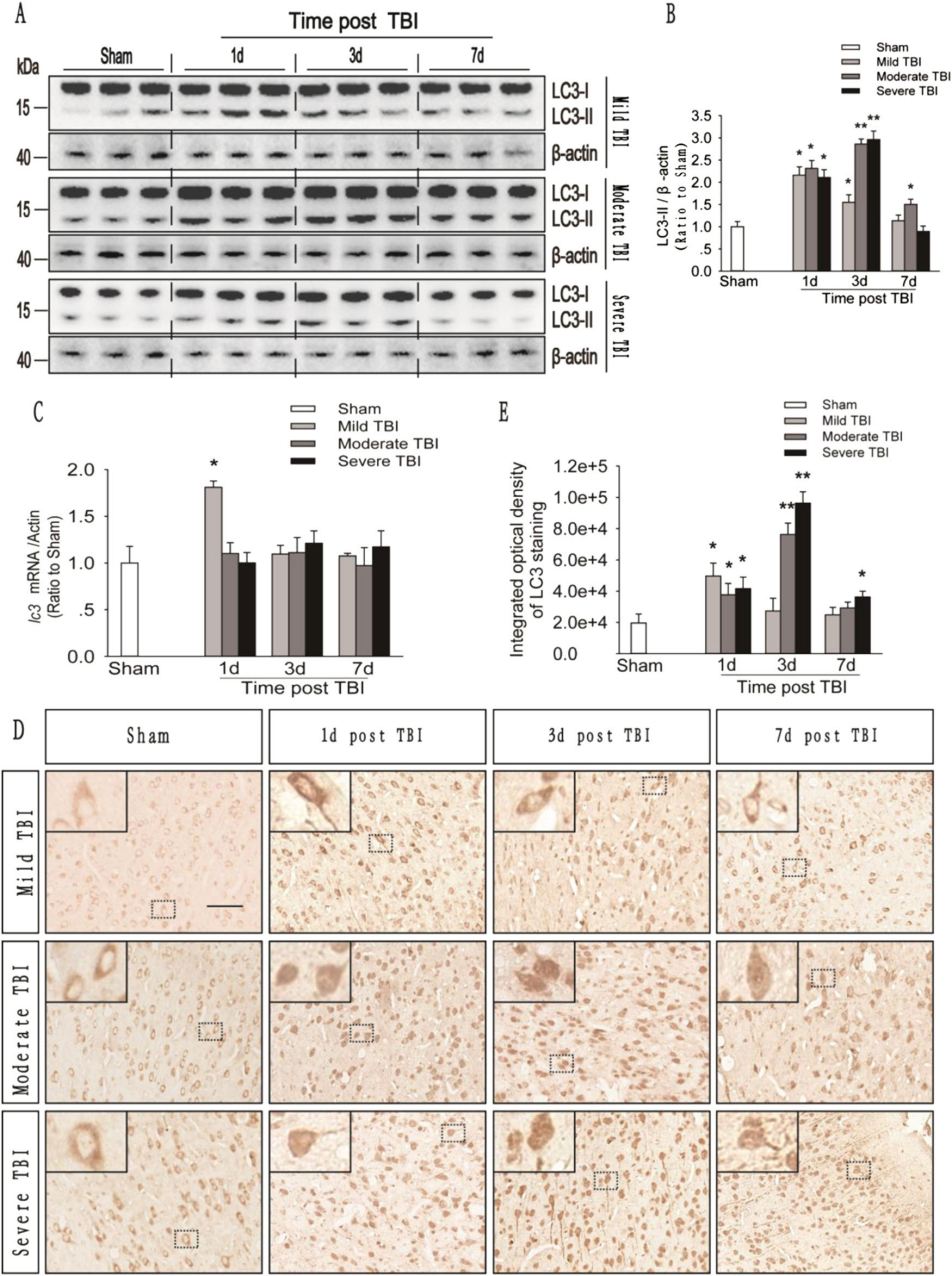
The number of autophagosomes in the injured cortex increases after mild, moderate and severe TBI. **a** Western blot analysis of the levels of the autophagy-related protein LC3 in cortical tissue lysates obtained from sham and TBI mice 1, 3, and 7 days after injury. **b** The LC3-II levels shown in (**a**) are quantified and normalized to the β-actin. The data shown are presented as means ± SEM, *n* = 5–6, *P < 0.05 and **P < 0.01. **c** Relative mRNA levels (qPCR) of *lc3* in the sham and injured mouse cortex. The results are normalized to β-actin levels. The data are presented as means ± SEM, *n* = 3, *P < 0.05 compared to the sham group. **d** Images of cortical brain sections obtained from sham and TBI mice. Sections were stained using an anti-LC3 antibody. Scale bar = 50 μm. **e** Integrated optical density analysis of the LC3 data shown in (**d**). The data are presented as means ± SEM, *n* = 5–6, *P < 0.05, **P < 0.01 compared to the sham group

The autophagy-related protein microtubule-associated protein 1 light chain 3b (MAP1LC3B/LC3) is cleaved and lipidated to form LC3-II, an important marker of autophagosomes^19–21^. Western blot analysis showed time-dependent increases in LC3-II levels in animals with mild, moderate, and severe injuries. In mice with a mid injury, LC3-II levels peaked before day 1 and then gradually decreased from days 3 to 7, whereas in mice with moderate or severe injury, LC3-II levels peaked between days 1 and 3, and then gradually decreased until day 7 (Fig. 1a, b). Consistent with these results, *Map1lc3b* mRNA levels in the cortex in animals with mild injury were significantly higher than those in sham animals on day 1 and then returned to normal levels from days 3 to 7. However, no substantial changes in *Map1lc3b* mRNA levels were observed in the animals with moderate or severe injury (Fig. 1c). In addition, immunohistochemistry revealed similar changes in levels of the LC3 protein among animals with mild, moderate, and severe injury (Fig. 1d, e). These results suggest that autophagosomes accumulate after mild, moderate, and severe trauma.

**Fig. 2:**
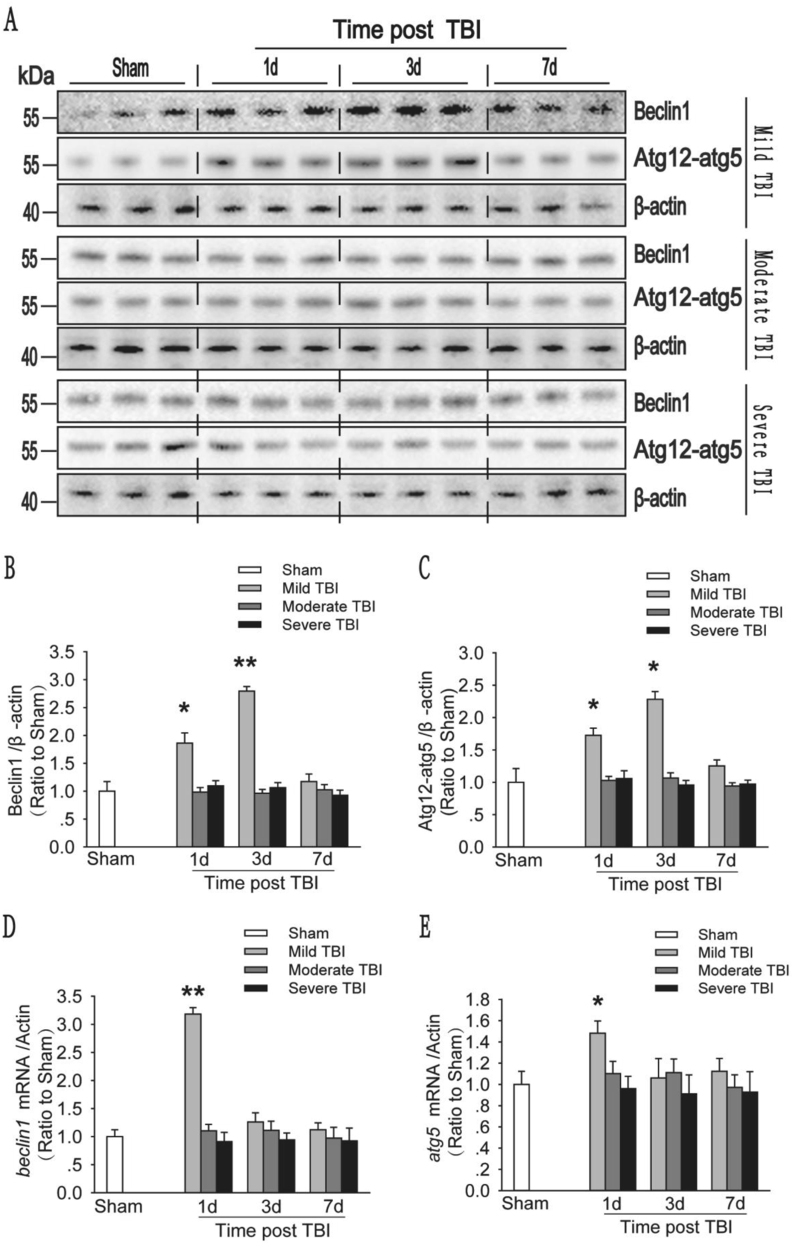
The initiation of autophagy is increased in response to mild injury, but shows no substantial change in response to moderate and severe injury. **a** Western blot analysis of the levels of the autophagy-related protein Beclin1 and the ATG12–ATG5 conjugate. **b** The Beclin1 levels shown in (**a**) are quantified and normalized to the β-actin levels. The data are presented as means ± SEM, *n* = 5–6, *P < 0.05. **c** The ATG12–ATG5 conjugate levels shown in (**a**) are quantified and normalized to the β-actin levels. The data are presented as means ± SEM, *n* = 5–6, *P < 0.05. **d** Relative mRNA levels (qPCR) of *beclin1* in the sham and injured mouse cortex. The results are normalized to β-actin levels. The data shown are presented as means ± SEM, *n* = 3, **P < 0.01 compared to the sham group. **e** Relative mRNA levels (qPCR) of *atg5* in the sham and injured mouse cortex. The results are normalized to β-actin levels. The data are presented as means ± SEM, *n* = 3, *P < 0.05 compared to the sham group

The autophagy-related protein Beclin1 and the autophagy-related protein 12 (ATG12)–ATG5 complex are markers of the initiation and elongation of autophagic flux^19^. We found that the protein levels of Beclin1 and the ATG12–ATG5 conjugate in animals with mild injury were markedly increased on days 1 and 3 compared with sham controls and then gradually decreased to a normal level by day 7. The mRNA levels of *beclin1* and *atg5* were also dramatically increased in these animals on day 1 and then decreased to a normal level on days 3 and 7. However, in the moderate and severe injury groups, there was no significant increase in the protein levels of Beclin1 or the ATG12–ATG5 conjugate or the mRNA levels of *beclin1* and *atg5* (Fig. 2a–e). The protein levels of Beclin1 and the ATG12–ATG5 conjugate, and the mRNA levels of *beclin1* and *atg5* are therefore altered by the severity of trauma following TBI. These results suggest that the initiation of autophagy is increased by mild TBI but not moderate or severe TBI.

**Fig. 3:**
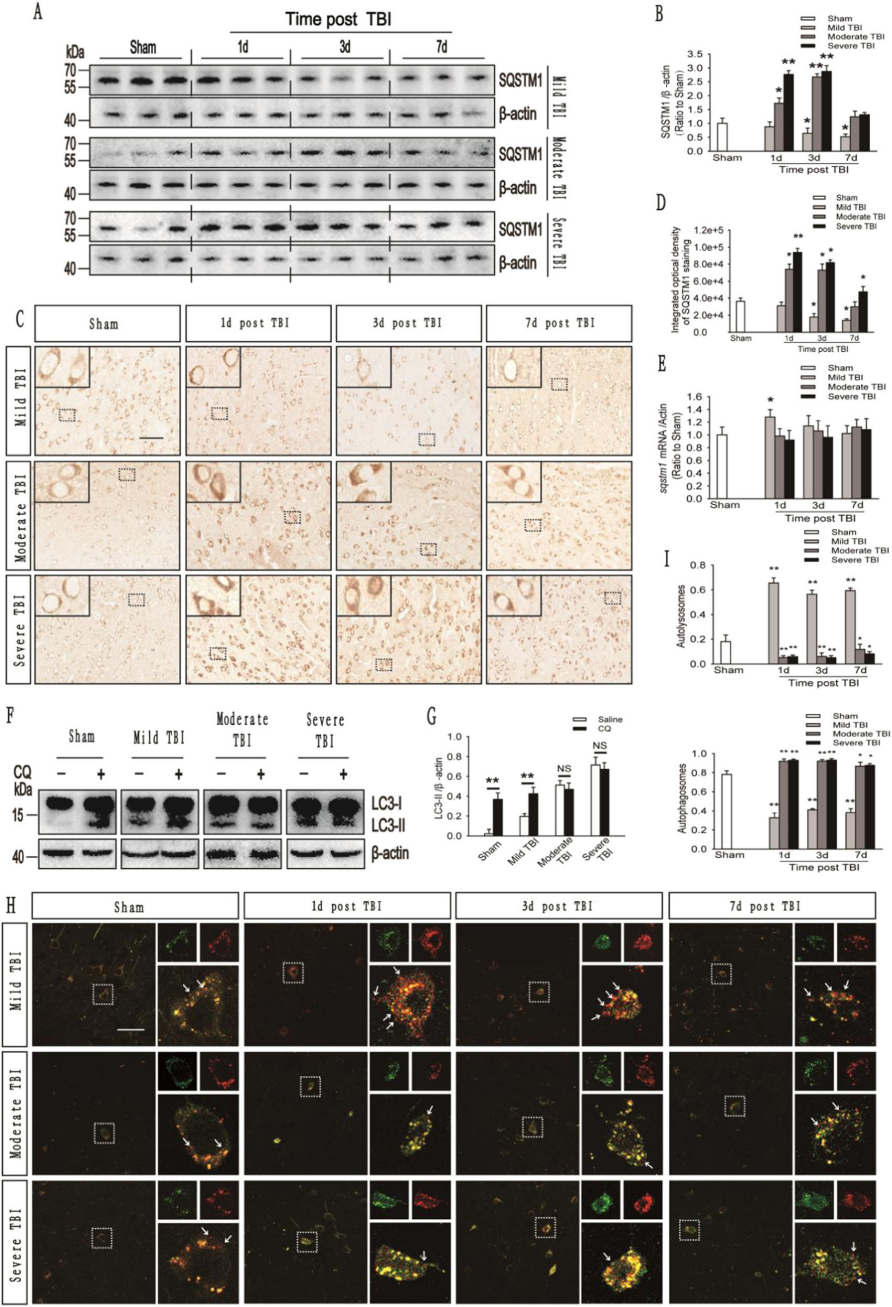
Autophagic flux is impaired in the cortex after moderate and severe TBI but not mild TBI. **a** Western blot analysis of SQSTM1 levels in cortical tissue lysates obtained from sham and TBI mice. **b** The SQSTM1 levels shown in (**a**) are quantified and normalized to the β-actin levels. The data are presented as means ± SEM, *n* = 5–6, *P < 0.05, **P < 0.01. **c** Images of cortical brain sections obtained from sham and TBI mice stained with antibodies against SQSTM1. Scale bar = 50 μm. **d** Integrated optical density analysis of the SQSTM1 data are shown in (**c**). The data are presented as means ± SEM, *n* = 5–6, *P < 0.05, **P < 0.01 compared to the sham group. **e** Relative mRNA levels (qPCR) of *sqstm1* in sham and TBI mice. The results are normalized to β-actin levels. The data are presented as means ± SEM, *n* = 3, *P < 0.05 compared to the sham group. **f** Western blot analysis of LC3 levels in the ipsilateral cortex of CQ-treated mice in the sham and TBI groups. **g** The LC3-II levels shown in **(f)** are quantified and normalized to the β-actin levels. The data are presented as means ± SEM, *n* = 3, **P < 0.01. **h** Images of cortical brain sections obtained from sham and TBI mice injected with AAV-mRFP-GFP-LC3. Arrows indicate the presence of red puncta. Scale bar = 40 μm. **i** Quantification of the percentage of autolysosomes (red puncta/total puncta) and autophagosomes (yellow puncta/total puncta) in the images shown in (**h**). The data are presented as means ± SEM, *n* = 3, *P < 0.05, **P < 0.01 compared to the sham group. More than 100 cells were quantified for each mouse in each experiment

Ubiquitinated cargoes, which include injured organelles and potentially toxic protein aggregates, are delivered to autophagosomes by the receptor protein sequestosome 1 (SQSTM1), which targets the cargoes along with other autophagy substrates for degradation by autolysosomes^22–24^. Hence, an increase in the SQSTM1 level reflects a decrease in autolysosomal functions. The results of immunohistochemistry and western blot assays revealed gradual reductions in the level of the SQSTM1 protein on days 1, 3, and 7 after mild TBI, whereas SQSTM1 levels markedly increased on days 1 through 3 after moderate or severe TBI and then gradually decreased through day 7 (Fig. 3a–d). Moreover, in animals with mild TBI, the mRNA level of *sqstm1* was slightly increased on day 1 after mild injury but was not significantly increased on days 3 and 7. However, no substantial change in the mRNA level of *sqstm1* was observed at any time point following moderate or severe TBI (Fig. 3e). The pH within a lysosome can be increased by chloroquine (CQ), and CQ treatment inhibits lysosomal degradation functions^25^. We performed a LC3-II turnover experiment to directly analyze whether autophagic flux is altered following mild, moderate, and severe TBI. Two hours after CQ administration, the protein level of LC3-II in the injured cortex was significantly increased in the mild TBI group, but not the moderate and severe TBI groups, compared with the saline-treated group (vehicle-treated group) (Fig. 3f, g). To further determine the effect of different severities of trauma on autophagic flux, we stereotactically injected adeno-associated virus (AAV)-mRFP-GFP-LC3 into the mouse cortex to create a mRFP-GFP-LC3-mouse model. Then, these mice were subjected to TBI 3 weeks after the injection. The GFP signal is sensitive to the acidic conditions of the lysosome lumen, whereas mRFP is more stable. Therefore, yellow puncta formed by the colocalization of GFP and mRFP fluorescence indicate autophagosomes, whereas red puncta denote autolysosomes. The percentage of red puncta was significantly increased, while the percentage of yellow puncta was decreased on days 1, 3 and 7 in the mild-injury group compared with the sham group. However, in the moderate- and severe-injury groups, the differences in the percentages of red and yellow puncta were reversed (Fig. 3h, i).These data confirm that autophagosome clearance is impaired after moderate and severe TBI.

These results collectively indicate that autophagic flux is impaired after moderate and severe TBI but not mild TBI.

### CQ impairs autophagic flux in the injured cortex and exacerbates the prognosis of mild TBI-induced brain injury

**Fig. 4:**
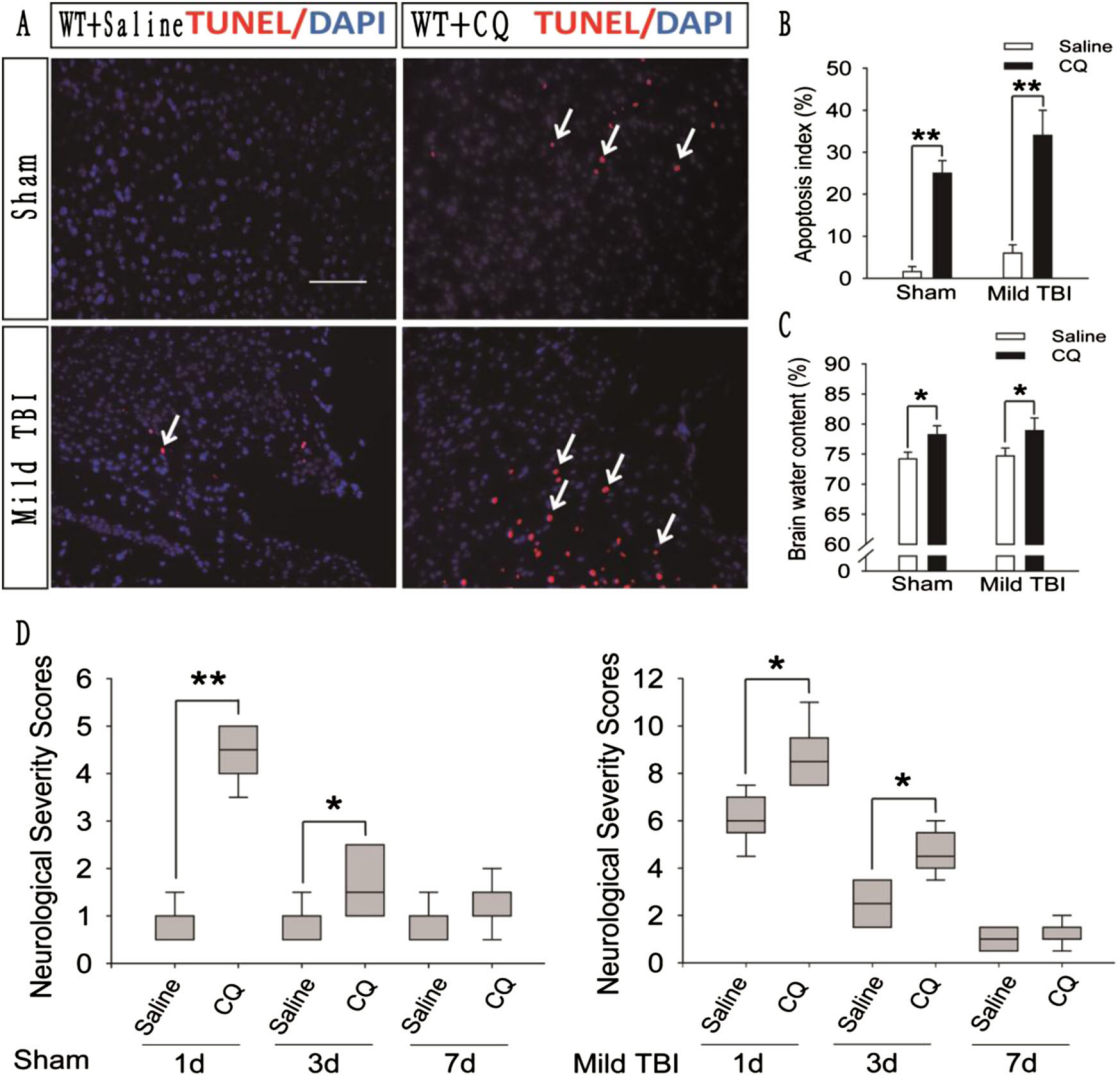
CQ impairs autophagic flux in the injured cortex of WT mice after mild TBI and exacerbates the prognosis of brain injury. **a** Images of apoptotic cells in cortical brain sections obtained from WT mice subjected to sham surgery or mild TBI that were administered CQ or saline. The results of TUNEL staining performed 1 day after sham surgery or mild TBI are shown. Arrows indicate TUNEL-positive cells. Scale bar = 50 μm. **b** Quantification of the number of TUNEL-positive cells in the cortical brain sections are shown in (**a**). The data are presented as means ± SEM, *n* = 5–6, **P < 0.01 compared to the control group. **c** Brain water content of WT mice 1 day after mice were subjected to sham surgery or mild TBI and then administered CQ or saline. The results were obtained using the wet–dry method. The data are presented as means ± SEM, *n* = 3, *P < 0.05 compared to the control group. **d** Neurological severity scores in WT mice subjected to sham surgery or mild TBI and then administered CQ or saline. Scores were obtained at 1, 3, and 7 days after sham surgery or mild TBI; the medians for all subjects are shown as the center line, the boxes represent the 25th–75th percentiles, and the lines show the range of the data, *n* = 7, *P < 0.05, **P < 0.01 compared to the control group at each time point

To explore the influence of impaired autophagic flux on the prognosis of brain injury, we used CQ to block autophagic flux in the injured side of the brain in mice subjected to mild TBI. We then detected the number of apoptotic cells in the injured cortex, the brain water content, and the neurological severity score (NSS) in the CQ-treated and saline-treated groups. The number of apoptotic cells in the injured cortex (Fig. 4a, b), the brain water content (Fig. 4c), and the NSS (Fig. 4d) were higher in the CQ-treated group than in the saline-treated group. Thus, the prognosis of mild brain injury is exacerbated when CQ is used to impair autophagic flux.

### A_2A_R KO or inhibition improves the prognosis of brain injury after moderate TBI

**Fig. 5:**
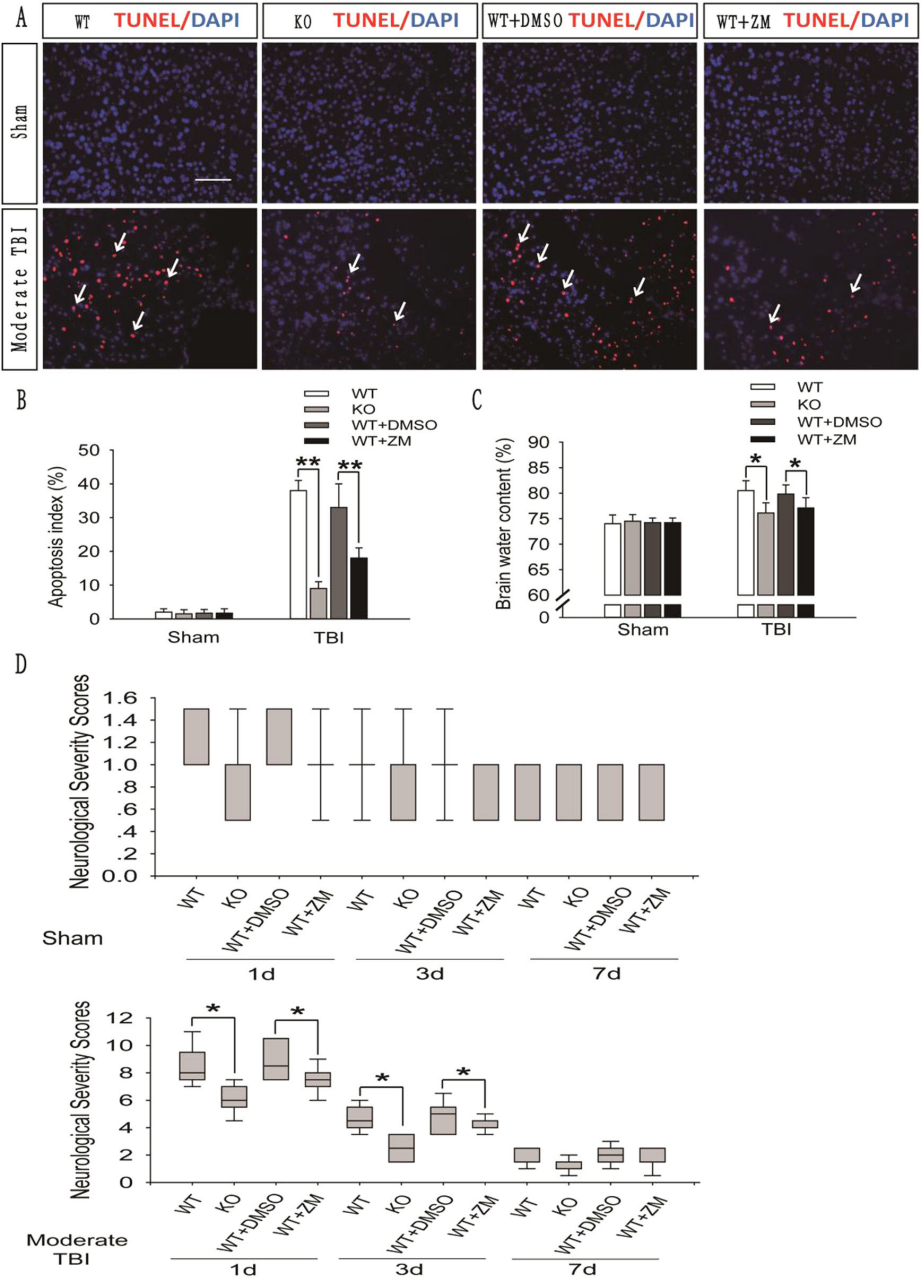
A_2A_R KO or inhibition improved the prognosis of brain injury after moderate TBI. **a** Images of apoptotic cells in cortical brain sections obtained from the WT + TBI, A_2A_R KO + TBI and WT + TBI + ZM241385 groups using TUNEL staining 1 day after sham surgery or moderate TBI. Arrows indicate TUNEL-positive cells. Scale bar = 50 μm. **b** Quantification of the number of TUNEL-positive cells in the cortical brain sections are shown in (**a**). The data are presented as means ± SEM, *n* = 3, **P < 0.01 compared to the WT + TBI group. **c** Brain water content in the WT + TBI, A_2A_R KO + TBI and WT + TBI + ZM241385 groups 1 day after sham surgery or moderate TBI. The results were measured using the wet–dry method. Th data are presented as means ± SEM, *n* = 3, *P < 0.05 compared to the WT + TBI group. **d** Neurological severity scores of the WT + TBI, A_2A_R KO + TBI and WT + TBI + ZM241385 groups at the indicated time points. Scores were obtained at 1, 3, and 7 days after sham surgery or moderate TBI; the medians for all subjects shown as the center lines, the boxes represent the 25th–75th percentile, and the lines show the range of the data, *n* = 7, *P < 0.05 compared to the WT + TBI group at each time point

Previous studies have shown that the presence of a large amount of adenosine induces the activation of A_2A_R after TBI^18, 26^. To determine whether A_2A_R activation exacerbates brain damage after moderate TBI, we counted the number of apoptotic cells in the injured cortex and measured the brain water content and NSS in WT and A_2A_R KO mice after inducing moderate TBI and in WT mice that were administered the A_2A_R antagonist 4-(2-{[5-amino-2-(2-furyl)[1,2,4]triazolo[1,5-a][1,3,5]triazin-7-yl]amino}ethyl)phenol (ZM241385) immediately after the induction of moderate TBI. The number of apoptotic cells in the injured cortex (Fig. 5a, b), the brain water content (Fig. 5c), and the NSS (Fig. 5d) were lower in the A_2A_R KO group than in the WT group and lower in the antagonist-administered group than in the DMSO-administered group (vehicle-administered group). Based on these results, A_2A_R KO or inhibition improved the prognosis of brain injury in mice with moderate TBI.

### Impaired autophagic flux is alleviated in the injured cortex of A_2A_R KO mice with moderate TBI

**Fig. 6:**
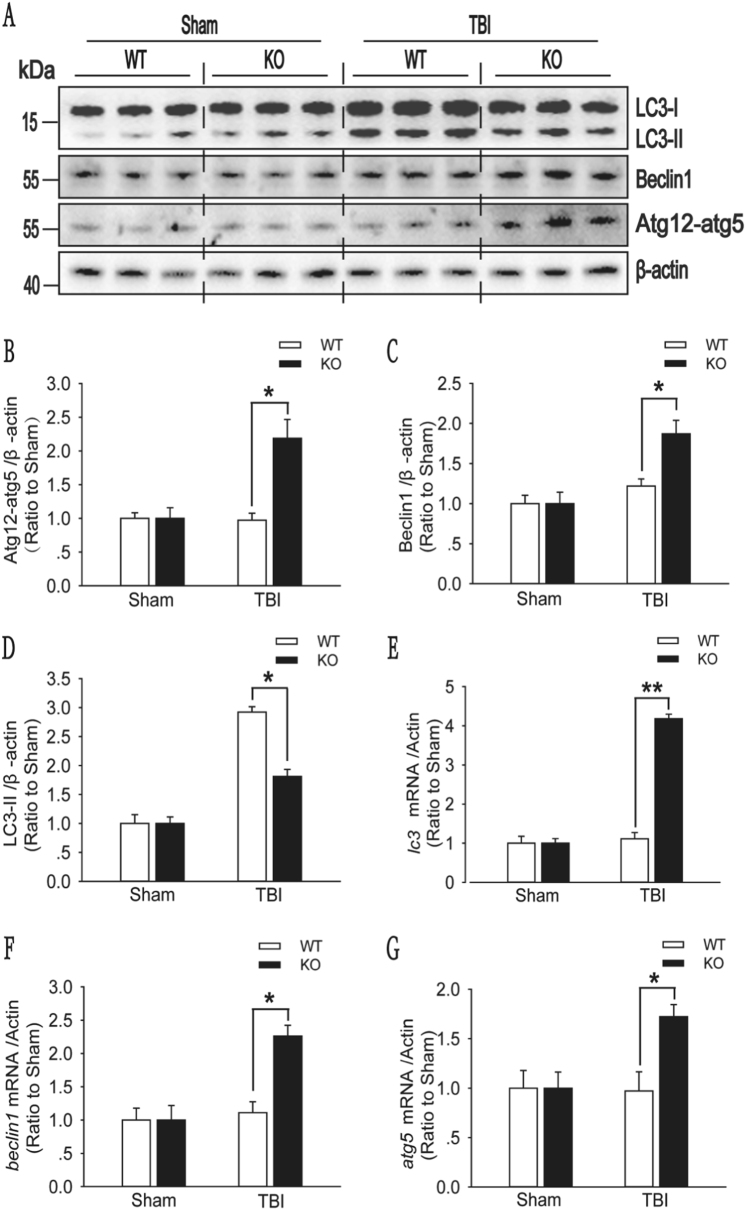
A_2A_R KO increased the initiation of autophagy and decreased the accumulation of autophagosomes in injured cortex after moderate TBI. **a** Western blot analysis of the levels of the autophagy-related protein Beclin1, the ATG12–ATG5 conjugate, and LC3 in cortical tissue lysates obtained from the sham and moderate TBI groups of WT and KO mice. **b** Beclin1 levels shown in (**a**) are quantified and normalized to β-actin levels. The data are presented as means ± SEM, *n* = 5–6, *P < 0.05. **c** ATG12–ATG5 conjugate levels shown in (**a**) are quantified and normalized to β-actin levels. Data are presented as means ± SEM, *n* = 5–6, *P < 0.05. **d** LC3-II levels shown in (**a**) are quantified and normalized to β-actin levels. The data are presented as means ± SEM, *n* = 5–6, **P < 0.01. **e** Relative mRNA levels (qPCR) of *beclin1* in sham and TBI mice are normalized to β-actin levels. The data are presented as means ± SEM, *n* = 3, *P < 0.05 for the comparison between the WT + TBI and KO + TBI groups. **f** Relative mRNA levels (qPCR) of *atg5* in sham and TBI mice are normalized to β-actin levels. The data are presented as means ± SEM, *n* = 3, *P < 0.05 for the comparison between the WT + TBI and KO + TBI groups. **g** Relative mRNA levels (qPCR) of *lc3* in sham and TBI mice are normalized to β-actin levels. The data are presented as means ± SEM, *n* = 3, **P < 0.01 for the comparison between the WT + TBI and KO + TBI groups

Western blot analysis showed increased protein levels of Beclin1 and the ATG12–ATG5 conjugate in the injured cortex of A_2A_R KO mice with moderate TBI (Fig. 6a–c), and the LC3-II level was markedly reduced (Fig. 6a, d). However, significantly higher mRNA levels of *beclin1*, *atg5* and *lc3* were observed in A_2A_R KO mice than in WT mice (Fig. 6e–g). These results suggest that A_2A_R KO increased the initiation of autophagy and decreased the accumulation of autophagosomes in the injured cortex.

**Fig. 7:**
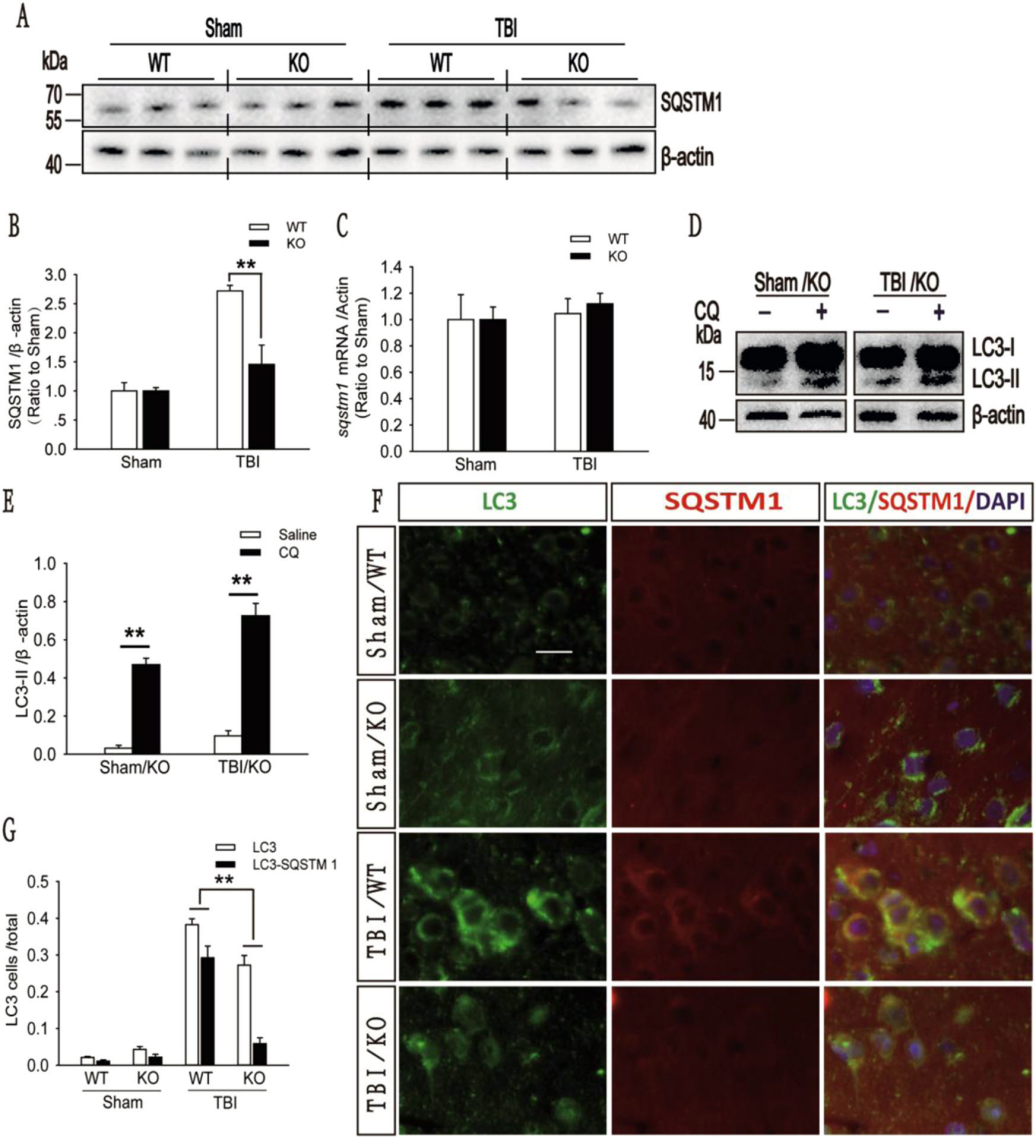
A_2A_R KO improved impaired fusion of autophagosomes with lysosomes. **a** Western blot analysis of SQSTM1 levels in cortical tissue lysates from the sham and moderate TBI groups of WT and KO mice. **b** SQSTM1 levels shown in (**a**) are quantified and normalized to β-actin levels. The data are presented as means ± SEM, *n* = 5–6, **P < 0.01. **c** Relative mRNA levels (qPCR) of *sqstm1* in sham and TBI mice are normalized to β-actin levels. The data are presented as means ± SEM, *n* = 3. **d** Western blot analysis of LC3 levels in the ipsilateral cortex of CQ- or saline-treated KO mice in the sham and moderate TBI groups. **e** LC3-II levels shown in (**d**) are quantified and normalized to β-actin levels. The data are presented as means ± SEM, *n* = 3, **P < 0.01. **f** Images (×20) of sections of the injured cortex from WT and KO mice in the sham and moderate TBI groups. Sections were stained with antibodies against LC3 and SQSTM1. Scale bar = 10 μm. **g** Quantification of the numbers of cells that were positive for LC3 alone and for both LC3 and SQSTM1 in the cortical brain sections shown in (**f**). The data are presented as means ± SEM, *n* = 3, **P < 0.01 for the comparison between the KO + TBI and WT + TBI groups. More than 1000 cells were quantified for each mouse in each experiment

Moreover, in the injured cortex, a significantly lower protein level of SQSTM1 was observed in A_2A_R KO mice than in WT mice (Fig. 7a, b), but the mRNA level of *sqstm1* was not significantly altered (Fig. 7c). In the LC3-II turnover experiment, the protein level of LC3-II was significantly increased in the ipsilateral cortex of both the sham and A_2A_R KO mice with moderate TBI 2 h after CQ treatment (Fig. 7d, e), but no significant increase was observed in the WT mice with moderate TBI compared with the saline-treated mice (Fig. 3f, g). The results of immunofluorescence assays also revealed that fewer cells from the injured cortex of A_2A_R KO mice displayed colocalization of LC3 and SQSTM1 than cells from the injured cortex of WT mice (Fig. 7f, g). These data confirm that A_2A_R KO increases autophagosome clearance in the injured cortex following moderate TBI.

## Discussion

### The presence of an impairment in autophagic flux in the injured cortex after TBI depends on the severity of trauma

Although many studies have shown that the prognosis of TBI is improved by strategies regulating autophagy, this finding remains controversial. Various authors have proposed that beneficial effects of treatments designed to enhance or inhibit autophagy after TBI^7–15^. Our data demonstrate that the protein levels of the Beclin1 and the ATG12–ATG5 conjugate, which are involved in the initiation and elongation of autophagy, and of LC3-II, which is closely related to the number of autophagosomes in a cell, are substantially increased in the injured cortex after mild TBI. The mRNA levels of *beclin1*, *atg5* and *lc3* were consistent with the observed protein levels of Beclin1, the ATG12–ATG5 conjugate and LC3-II. However, the level of the SQSTM1 protein, an indicator of autolysosomal function, was reduced. In addition, we administered CQ, which increases the pH within the lysosome, to the injured cortex of animals with mild TBI and found that CQ impaired autophagic flux. A significantly higher LC3-II level was detected in the CQ-treated group than in the saline-treated group. Moreover, in the autophagic flux assay, we observed an increase in the percentage of red puncta and a decrease in the percentage of yellow puncta, indicating an increase in the percentage of autolysosomes and a decrease in the percentage of autophagosomes after mild injury. These data demonstrate that, while autophagic flux was normal following mild TBI, the initiation of autophagy and the formation of autolysosomes were enhanced. In moderate and severe TBI, no substantial changes in the levels of the Beclin1 protein and the ATG12–ATG5 conjugate were observed. Additionally, the LC3-II and SQSTM1 levels were markedly increased, but no clear increase in the mRNA levels of *beclin1*, *atg5*, *lc3* and *sqstm1* was detected. Moreover, in the LC3-II turnover experiment, the levels of LC3-II protein did not differ between the saline-treated animals and the CQ-treated animals and in autophagic flux assay, we observed a reduction in the percentage of autolysosomes and an increase in the percentage of autophagosomes after moderate and severe TBI. These data reveal that in animals with moderate and severe TBI, the initiation of autophagy is not changed, and autophagosomes subsequently accumulate in cells because autophagic flux is impaired. The increased number of autophagosomes negatively impacts prognosis of brain injury because autophagy itself is attenuated. A previous study published by Sarkar et al.^16^ showed that autophagic flux was impaired after TBI, but the authors did not discuss the conditions or causes associated with this occurrence. Therefore, it remains difficult to explain why many studies have found that enhancing the initiation of autophagy during the early stage after TBI is beneficial to brain injury prognosis^13, 27–31^. Our data indicate that autophagic flux is impaired after moderate and severe TBI but not mild TBI. It is therefore reasonable to treat patients differently according to the severity of trauma associated with their TBI. In a patient with a mild injury, the prognosis of brain injury will improve when the initiation of autophagy is enhanced because autophagic flux is normal. However, when autophagic flux is impaired, as in patients with moderate and severe TBI, strategies that inhibit the initiation of autophagy will reduce the accumulation of autophagosomes and promote the fusion of autophagosomes with lysosomes, accelerating autophagosome clearance and subsequently reducing brain damage.

### Impaired autophagic flux exacerbates the severity of trauma

In our study, we used CQ to impair the autophagic flux in the injured cortex after mild TBI and found that the number of apoptotic brain cells, the brain water content, and NSS were higher in the CQ-treated group than in the saline-treated group. These results indicate that the prognosis of mild brain injury is exacerbated when CQ is used to impair autophagic flux. Therefore, treatments that alleviate the severity of trauma by alleviating the impairment in autophagic flux after TBI would be beneficial. Although some methods designed to regulate autophagy are currently available, treatments that improve impaired autophagic flux are still lacking. Thus, we next explored the mechanism of impaired autophagic flux after TBI.

### A_2A_R KO enhances the ability of cells to avoid injury by alleviating the impairments in autophagic flux in animals with moderate TBI

In the present study, we observed higher protein levels of Beclin1 and the ATG12–ATG5 conjugate, lower protein levels of LC3-II and SQSTM1 and higher mRNA levels of *beclin1*, *atg5*, and *lc3* in the injured cortex of A_2A_R KO mice than in that of WT mice. No significant difference in the mRNA level of *sqstm1* was observed between the two groups. In addition, in an LC3-II turnover experiment, significantly higher LC3-II levels were detected in the injured cortex 2 h after CQ administration than in the injured cortex after saline treatment in A_2A_R KO mice. These results indicate that A_2A_R KO alleviated the impairment in autophagic flux by enhancing the initiation of autophagy and promoting fusion between autophagosomes and lysosomes in the injured cortex after moderate TBI. A_2A_R KO thereby prevented the accumulation of toxic metabolites and consequently improved recovery following brain damage. Although it has been confirmed that inhibition of A_2A_R reduces cell damage through a variety of ways^32–37^. In our experiment, we used CQ to impair autophagic flux in the injured cortex after mild TBI and found that the prognosis of mild brain injury was worsened when CQ was used to impair autophagic flux. However, this exacerbating effect was significantly reduced in A_2A_R KO mice **(**Fig. S1A–D**)**. Thus, the A_2A_R inhibition-mediated alleviation of the impairment in autophagic flux is an important mechanism that reduces cell damage and decreases injury severity from TBI.

Previous studies have reported that the activation of A_2A_R reduces autophagy functions^38, 39^ and that the level of phosphorylated tau protein and the accumulation of amyloid plaques are reduced in A_2A_R KO mice^40^. In recent years, dysfunctional autophagy has been implicated in neuronal cell loss in neurodegenerative diseases, such as Alzheimer’s disease and Parkinson’s disease^41–43^, and the transcription factor EB, a major regulator of autophagy and lysosome biogenesis, has emerged as a leading therapeutic factor in the pathology of these neurodegenerative diseases^43^. Settembre et al. ^44^. showed that ERK2 regulates the phosphorylation of transcription factor EB by studying the relationship between transcription factor EB and autophagy, ERK2 is a component of the classical A_2A_R pathway. Thus, A_2A_R may impair autophagic flux via an ERK2/ transcription factor EB pathway. However, the specific mechanism remains to be further confirmed.

Although many methods have been used to regulate autophagy, no methods designed to improve impaired autophagic flux are currently available. Here, we provide the first data showing that A_2A_R KO improved impaired autophagic flux by enhancing the initiation of autophagy and promoting the fusion of autophagosomes to lysosomes in mice with moderate TBI. A previous study suggested that primary brain cell death is inevitable after TBI. However, secondary brain cell death can be salvaged, and the key goal in these patients is to maintain a sufficiently high ATP level in brain cells^45^. Autophagy is an important physiological process that provides cells with energy and plays an important role in brain cell survival after TBI. Therefore, strategies that enhance the initiation of autophagy and promote normal autophagic flux are key to eventually enhancing autophagy itself. Hence, A_2A_R antagonists that modulate autophagy in brain cells after TBI may represent an effective treatment option.

### Conclusion

In summary, the number of autophagosomes increases after TBI, but whether autophagic flux is impaired determines whether autophagy exerts a protective effect against brain damage. Our findings provide critical evidence revealing significant differences in the influence of the severity of trauma on autophagic flux and show that A_2A_R activation may play an important role in impairing autophagic flux in response to moderate TBI. Therefore, any analysis of autophagic flux after TBI should consider the severity of trauma, and we propose that regulating A_2A_R is a promising strategy for treating moderate TBI because inhibiting A_2A_R alleviated the TBI-induced impairments in autophagic flux.

## Methods and Materials

### Animals

A_2A_R KO mice were established using gene targeting, as previously described. The mice used in this study were provided by Dr. Chen^40, 41^. Congenic global A_2A_R KO mice with a C57BL/6J background were generated by backcrossing global A_2A_R KO mice with a mixed (129-Steel × C57BL/6J) genetic background to C57BL/6J mice for 13–15 generations. Their littermates were used as WT mice in these experiments. The mice were fed in a pathogen-free room with controlled humidity and temperature, and housed under a 12-h light/dark cycle in the Animal Care Center of the Research Institute of Surgery and Daping Hospital (The Third Military Medical University, Chongqing, China). Male mice aged 8–12 weeks that weighed 22–26 g were used in our experiments. Mice were allocated to the sham, mild TBI, moderate TBI and severe TBI groups using a random number table. All animal procedures were reviewed and approved by the Administration of Affairs Concerning Experimental Animals Guidelines of The Third Military Medical University.

### TBI model

The controlled cortical impact method was used to produce mild, moderate, and severe TBI models according to the methods described in our previous protocol, which involve measuring brain water content and neurological deficit scores^46^. Briefly, mice were anesthetized with 1.5% pentobarbital sodium at a dose of 50 mg/kg and then subjected to a 4–5-mm-diameter craniotomy in the left parietal cortex using a motorized drill. The center was placed between bregma and the lambdoid suture. We used an aerodynamic impact device (BRAIN INJURY DEVICE TBI-0310, PSI, USA) with a metal tip with a 3-mm-diameter metal tip to produce the controlled cortical impact. We used the following parameters: 1 mm below the dura for the mild TBI model, 2 mm below the dura for the moderate TBI model and 4 mm below the dura for the severe TBI model at an impact speed of 3.5 m/s. After the wound was sutured, we used an electric heating blanket to maintain the animals’ body temperature until they were completely awake and able to move freely, which occurred approximately 3 h after the injury.

### Neurologic severity scoring and edema evaluation

Mice were scored for neurologic severity at 1, 3, and 7 days after TBI using the method described by Petullo, D et al. ^47^. Briefly, neuromuscular function, including forelimb flexion, torso twisting, lateral push, hindlimb placement, and forelimb placement; performance on an inclined board; mobility; vestibulomotor function, including performance on a balance beam; and complex neuromotor functions, including performance on a beam walk, were evaluated and given a score of 0–1 or 0–2 for neuromuscular functions, 0–6 for vestibulomotor functions and 0–5 for complex neuromotor functions. The brain water content was assayed using a wet–dry method at 3 days after TBI, as previously described^48^. Briefly, the ipsilateral cortices were immediately removed, weighed (wet weight) and placed in an 80 °C incubator for 48 h. Then, the cortices were weighed again (dry weight). The brain water content was calculated as a percentage as follows: [(wet weight – dry weight) / wet weight] × 100%.

### Immunohistochemistry, immunofluorescence and TUNEL assays

Mice were anesthetized using 1.5% pentobarbital sodium at a dose of 50 mg/kg and then sequentially perfused with saline and 4% paraformaldehyde. Brains were dissected from the calvarium and post-fixed with 4% paraformaldehyde. Coronal sections (5 μm-thick) were cut from paraffin-embedded tissues and prepared for immunohistochemistry, immunofluorescence and TUNEL assays. Sections were incubated with the following primary antibodies overnight at 4 °C: anti-LC3B (1:200; Abcam, ab64781, Cambridge, USA), anti-SQSTM1 (1:200; Abcam, ab91526), and anti-SQSTM1 (1:200; Abcam, ab56416). For immunofluorescence analyses, sections were rewarmed for 30 min, washed with PBS, and then incubated with Alexa Fluor 488-conjugated (1:200; Abcam, ab150077) and Cy3-conjugated secondary antibodies (1:200; Abcam, ab97035) for 1 h at room temperature. Sections were then washed and mounted on slides using UltraCruz^TM^ hard-set mounting medium with DAPI (Santa Cruz Biotechnology, sc-359850, Dallas, TX, USA). For immunohistochemical analyses, after the sections were incubated with secondary antibodies, a streptavidin/peroxidase and diaminobenzidine substrate kit (ZSGB-BIO, Beijing, China) was used to visualize the results. TUNEL assays were performed on paraffin-embedded brain sections using an In Situ Cell Death Detection Kit, TMR red (Roche, 12156792910, California, USA). The total number of brain cells and the number of TUNEL-positive cells were manually counted; the apoptotic index (AI) was defined as the percentage of TUNEL-positive cells among the total number of brain cells^49, 50^. We used previously described methods and Image-Pro Plus 4.5 software (Media Cybernetics, Rockville, MD, USA) to analyze the results^46, 51^. All measurements were obtained from one field in each of three slices per mouse.

### Real-time PCR

Mice were anesthetized using 1.5% pentobarbital sodium at a dose of 50 mg/kg, and the brains were dissected from the calvarium at 1, 3 and 7 days after the inducti-on of mild, moderate and severe TBI. Total RNA was isolated from the injured cortex using TRIzol (Invitrogen, 10296010, Carlsbad, CA, USA) and then reverse-transcrib-ed. A SYBR Green kit (TaKaRa Bio Inc., RR820L, Shiga, Japan) was used for quan-titative PCR. The following primers were used to measure the levels of the *beclin1*, *atg5*, *map-1lc3b* and *sqstm1* mRNAs: *beclin1* (forward: 5′-AGGAACTCACAGCTCCAT-TAC-3′ and reverse: 5′-AATGGCTCCTCTCCTGAGTT-3′), *atg5* (forward: 5′-TTCATCCAGAAGCTGTTCCG-3′ and reverse: 5′-ATTGGCTCTATCCCGTGAATC-3′), *map1lc3-b* (forward: 5′-ACA-AAGAGTGGAAGATGTCCG-3′ and reverse: 5′-CCCCTTGTATCG-CTCTATAATCAC-3′), and *sqstm1* (forward: 5′-CCTATACCCACATCTCCCACC-3′ and reverse: 5′-TGTCGTAATTCTTGGTCTGTAGG-3′).

### Western blot assays

Mice were anesthetized with 1.5% pentobarbital sodium at a dose of 50 mg/kg, perfused with ice-cold saline and then decapitated. Approximately 5 mm of the injured cortex was collected and homogenized in RIPA buffer (Thermo Fisher Scientific, 89901, Waltham, MA, USA) containing protease (Roche, 11836170001) and phosphatase (Sigma, P5726, St. Louis, MO, USA) inhibitors. Homogenates were centrifuged at 20,000 g for 20 min at 4 °C to collect the lysates. Protein concentrations were measured using BCA reagent (Thermo Fisher Scientific, 23225). Proteins (20 μg per sample) were resolved on 12% SDS-PAGE gels and then transferred to PVDF membranes (Millipore, IPVH00010, Massachusetts, USA). The Membranes were blocked with 5% nonfat milk and probed with the following primary antibodies overnight at 4 °C: anti-LC3B (1:1000; Sigma, L7543), anti-SQSTM1 (1:1000; Abcam, ab91526), anti-SQSTM1 (1:1000; Abcam, ab56416), anti-Beclin1 (1:1000, Santa Cruz Biotechnology, sc-11427), anti-Atg5 (1:1000; Abcam, ab78073), and anti-β-actin (1:1000; Abcam, ab8226). After an incubation with HRP-conjugated secondary antibodies, membranes were visualized using SuperSignal Chemiluminescent Substrates (Thermo Fisher Scientific, 34080).

### LC3 turnover assay in injured brains

Mice were anesthetized with 1.5% pentobarbital sodium at a dose of 50 mg/kg on day 3 after TBI. Then, mice were fixed in a brain stereotaxic apparatus (Stoelting, 51750, USA) and the distal end of the injection tube of a microinjection system (RWD, 62322, 62204, 62104, China) was placed (bregma: AP, −2 mm; ML, 1.0 mm; and DV, 0.5 mm) in the injured cortex. A pressure injection was then performed to inject 2 μL of CQ (100 μM; Sigma, C6628) at a speed of 0.1 μL/min using a microinjection pump (Stoelting, 53311, USA). The injection tube was maintained in position within the injured cortex for 20 min after the injection was complete, and the wound was then sutured. Finally, the mice were anesthetized, perfused with ice-cold saline, and decapitated. Approximately 5 mm of the cortical area surrounding the injured cortex was collected and homogenized in RIPA buffer (Thermo Fisher Scientific, 89901) containing protease (Roche, 11836170001) and phosphatase (Sigma, P5726) inhibitors. Homogenates were centrifuged at 20,000 g for 20 min at 4 °C to collect the tissue lysates. Protein concentrations were measured using BCA reagent (Thermo Fisher Scientific, 23225), and lysates were analyzed using Western blot.

### Autophagic flux measurement

AAV-mRFP-GFP-LC3 was purchased from Hanbio (Shanghai, China) and stereotactically injected into ipsilateral cortex of mouse (2 μL) before 3 weeks before TBI. The injection system used for the LC3 turnover assay is described above. Briefly, the mice were anesthetized with 1.5% pentobarbital sodium at a dose of 50 mg/kg and then sequentially perfused with saline and 4% paraformaldehyde. Brains were dissected from the calvarium and post-fixed with 4% paraformaldehyde. Coronal sections (30 μm) were cut and photographed with a laser confocal microscope (Leica TCS SP8, Frankfurt, Germany). The relative fluorescence intensity was analyzed with Image-Pro Plus 6.0 software (Media Cybernetics, Rockville, MD, USA). GFP, but not mRFP, degrades in an acidic environment. Thus, yellow spots (merge of red and green) indicate autophagosomes, whereas red spots indicate autolysosomes. If autophagy is activated and the autophagic flux is normal, the red signal will dominate over the yellow signal. Meanwhile, if autophagic flux is impaired, more yellow signal than red signal will be observed.

### ZM241385 injection

The injection system used for the LC3 turnover assay is described above. The A_2A_R antagonist ZM241385 (1 mg/kg) was administered into the injured cortex immediately after mild TBI was induced. Mice were anesthetized and processed for TUNEL assays and to evaluate edema after 3 days and scored for neurologic severity after 1, 3, and 7 days.

### Statistical analysis

All results are expressed as the means ± SEM. All semi-quantitative assessments of histological staining results were performed by a single investigator who was blinded to the genotypes of and treatments received by the experimental animals. Differences between two groups were analyzed using Student’s *t* test for data that passed the normality and equal variance tests or the rank sum test for discontinuous variables, and statistical comparisons of more than two groups were performed using a one-way ANOVA followed by Bonferroni’s post hoc test (parametric) or Kruskal–Wallis ANOVA followed by Dunn’s post hoc test (nonparametric). A value of *P* < 0.05 was considered statistically significant.

## Electronic supplementary material

Figure S1

Figure S1 legend

## References

[1] Klionsky DJ, Emr SD (2000). Autophagy as a regulated pathway of cellular degradation. Science.

[2] Mizushima N, Komatsu M (2011). Autophagy: renovation of cells and tissues. Cell.

[3] Klionsky DJ (2006). Neurodegeneration: good riddance to bad rubbish. Nature.

[4] Shintani T, Klionsky DJ (2004). Autophagy in health and disease: a double-edged sword. Science.

[5] Butler D, Nixon RA, Bahr BA (2006). Potential compensatory responses through autophagic/lysosomal pathways in neurodegenerative diseases. Autophagy.

[6] Nixon RA (2013). The role of autophagy in neurodegenerative disease. Nat. Med..

[7] Werner C, Engelhard K (2007). Pathophysiology of traumatic brain injury. Br. J. Anaesth..

[8] Clark RS (2008). Autophagy is increased in mice after traumatic brain injury and is detectable in human brain after trauma and critical illness. Autophagy.

[9] Diskin T (2005). Closed head injury induces upregulation of Beclin 1 at the cortical site of injury. J. Neurotrauma.

[10] Erlich S, Alexandrovich A, Shohami E, Pinkas-Kramarski R (2007). Rapamycin is a neuroprotective treatment for traumatic brain injury. Neurobiol. Dis..

[11] Luo CL (2011). Autophagy is involved in traumatic brain injury-induced cell death and contributes to functional outcome deficits in mice. Neuroscience.

[12] Wang YQ (2012). Necrostatin-1 suppresses autophagy and apoptosis in mice traumatic brain injury model. Neurochem. Res..

[13] Liu CL, Chen S, Dietrich D, Hu BR (2008). Changes in autophagy after traumatic brain injury. J. Cereb. Blood. Flow. Metab..

[14] Bao, H. J., Zhang, L., Han, W. C. & Dai, D. K. Apelin-13 attenuates traumatic brain injury-induced damage by suppressing autophagy. *Neurochem. Res.* **40**, 29–97 (2014). 10.1007/s11064-014-1469-x 25362565

[15] Zhang M (2014). Hydrogen sulfide offers neuroprotection on traumatic brain injury in parallel with reduced apoptosis and autophagy in mice. PLoS. One..

[16] Sarkar, C. et al. Impaired autophagy flux is associated with neuronal cell death after traumatic brain injury. *Autophagy* **10**, 2208–2222 (2014). 10.4161/15548627.2014.981787 PMC4502690 25484084

[17] Lai Y (2008). Autophagy is increased after traumatic brain injury in mice and is partially inhibited by the antioxidant gamma-glutamylcysteinyl ethyl ester. J. Cereb. Blood. Flow. Metab..

[18] Chen JF, Lee CF, Chern Y (2014). Adenosine receptor neurobiology: overview. Int. Rev. Neurobiol..

[19] Mizushima N, Yoshimori T, Ohsumi Y (2011). The role of Atg proteins in autophagosome formation. Annu. Rev. Cell. Dev. Biol..

[20] Kabeya Y (2000). LC3, a mammalian homologue of yeast Apg8p, is localized in autophagosome membranes after processing. Embo. J..

[21] Ichimura Y (2000). A ubiquitin-like system mediates protein lipidation. Nature.

[22] Pankiv S (2007). p62/SQSTM1 binds directly to Atg8/LC3 to facilitate degradation of ubiquitinated protein aggregates by autophagy. J. Biol. Chem..

[23] Bjorkoy G, Lamark T, Johansen T (2006). p62/SQSTM1: a missing link between protein aggregates and the autophagy machinery. Autophagy.

[24] Ichimura Y, Kominami E, Tanaka K, Komatsu M (2008). Selective turnover of p62/A170/SQSTM1 by autophagy. Autophagy.

[25] Klionsky DJ (2016). Guidelines for the use and interpretation of assays for monitoring autophagy (3rd edition. Autophagy.

[26] Li W (2009). Genetic inactivation of adenosine A2A receptors attenuates acute traumatic brain injury in the mouse cortical impact model. Exp. Neurol..

[27] Zhao M, Liang F, Xu H, Yan W, Zhang J (2016). Methylene blue exerts a neuroprotective effect against traumatic brain injury by promoting autophagy and inhibiting microglial activation. Mol. Med Rep..

[28] Zhang L, Ding K, Wang H, Wu Y, Xu J (2016). Traumatic Brain Injury-Induced Neuronal Apoptosis is Reduced Through Modulation of PI3K and Autophagy Pathways in Mouse by FTY720. Cell. Mol. Neurobiol..

[29] Gao Y (2016). Tetrahydrocurcumin provides neuroprotection in rats after traumatic brain injury: autophagy and the PI3K/AKT pathways as a potential mechanism. J. Surg. Res..

[30] Ding K (2015). Melatonin protects the brain from apoptosis by enhancement of autophagy after traumatic brain injury in mice. Neurochem. Int..

[31] Zhang YB (2008). Autophagy is activated and might protect neurons from degeneration after traumatic brain injury. Neurosci. Bull..

[32] Pierri M, Vaudano E, Sager T, Englund U (2005). KW-6002 protects from MPTP induced dopaminergic toxicity in the mouse. Neuropharmacology.

[33] Simoes AP (2012). Blockade of adenosine A2A receptors prevents interleukin-1beta-induced exacerbation of neuronal toxicity through a p38 mitogen-activated protein kinase pathway. J. Neuroinflamm..

[34] Alfinito PD (2003). Adenosinergic protection of dopaminergic and GABAergic neurons against mitochondrial inhibition through receptors located in the substantia nigra and striatum, respectively. J. Neurosci..

[35] Popoli P (2002). Blockade of striatal adenosine A2A receptor reduces, through a presynaptic mechanism, quinolinic acid-induced excitotoxicity: possible relevance to neuroprotective interventions in neurodegenerative diseases of the striatum. J. Neurosci..

[36] Dall’Igna OP, Porciuncula LO, Souza DO, Cunha RA, Lara DR (2003). Neuroprotection by caffeine and adenosine A2A receptor blockade of beta-amyloid neurotoxicity. Br. J. Pharmacol..

[37] Cunha GM, Canas PM, Oliveira CR, Cunha RA (2006). Increased density and synapto-protective effect of adenosine A2A receptors upon sub-chronic restraint stress. Neuroscience.

[38] Ke, J., Yao, B., Li, T., Cui, S. & Ding, H. A2 adenosine receptor-mediated cardioprotection against reperfusion injury in rat hearts is associated with autophagy down-regulation. *J. Cardiovasc. Pharmacol*. **66**, 25–34 (2015). 10.1097/FJC.0000000000000239 25706370

[39] Liu YW (2016). Activation of Adenosine 2A receptor inhibits neutrophil apoptosis in an autophagy-dependent manner in mice with systemic inflammatory response syndrome. Sci. Rep..

[40] Laurent C (2016). A2A adenosine receptor deletion is protective in a mouse model of Tauopathy. Mol. Psychiatry.

[41] Wolfe DM (2013). Autophagy failure in Alzheimer’s disease and the role of defective lysosomal acidification. Eur. J. Neurosci..

[42] Nixon RA, Yang DS (2011). Autophagy failure in Alzheimer’s disease--locating the primary defect. Neurobiol. Dis..

[43] Martini-Stoica H, Xu Y, Ballabio A, Zheng H (2016). The autophagy-lysosomal pathway in neurodegeneration: a TFEB perspective. Trends Neurosci..

[44] Settembre C (2011). TFEB links autophagy to lysosomal biogenesis. Science.

[45] Ankarcrona M (1995). Glutamate-induced neuronal death: a succession of necrosis or apoptosis depending on mitochondrial function. Neuron.

[46] Li W (2008). Chronic but not acute treatment with caffeine attenuates traumatic brain injury in the mouse cortical impact model. Neuroscience.

[47] Petullo D (1999). Model development and behavioral assessment of focal cerebral ischemia in rats. Life. Sci..

[48] Okiyama K (1995). The sodium channel blocker and glutamate release inhibitor BW1003C87 and magnesium attenuate regional cerebral edema following experimental brain injury in the rat. J. Neurochem..

[49] Shao L (2015). SENP1-mediated NEMO deSUMOylation in adipocytes limits inflammatory responses and type-1 diabetes progression. Nat. Commun..

[50] Hu Q, Luo W, Huang L, Huang R, Chen R (2016). Apoptosis-related microRNA changes in the right atrium induced by remote ischemic perconditioning during valve replacement surgery. Sci. Rep..

[51] Dai SS (2013). Plasma glutamate-modulated interaction of A2AR and mGluR5 on BMDCs aggravates traumatic brain injury-induced acute lung injury. J. Exp. Med..

