# Supplementary figures and images for: Impaired autophagic flux is associated with the severity of trauma and the role of A2AR in brain cells after traumatic brain injury
Source: Cell Death Dis. 2018 Feb 14;9(2):252. doi: 10.1038/s41419-018-0316-4 (PMC5833790; doi:10.1038/s41419-018-0316-4)

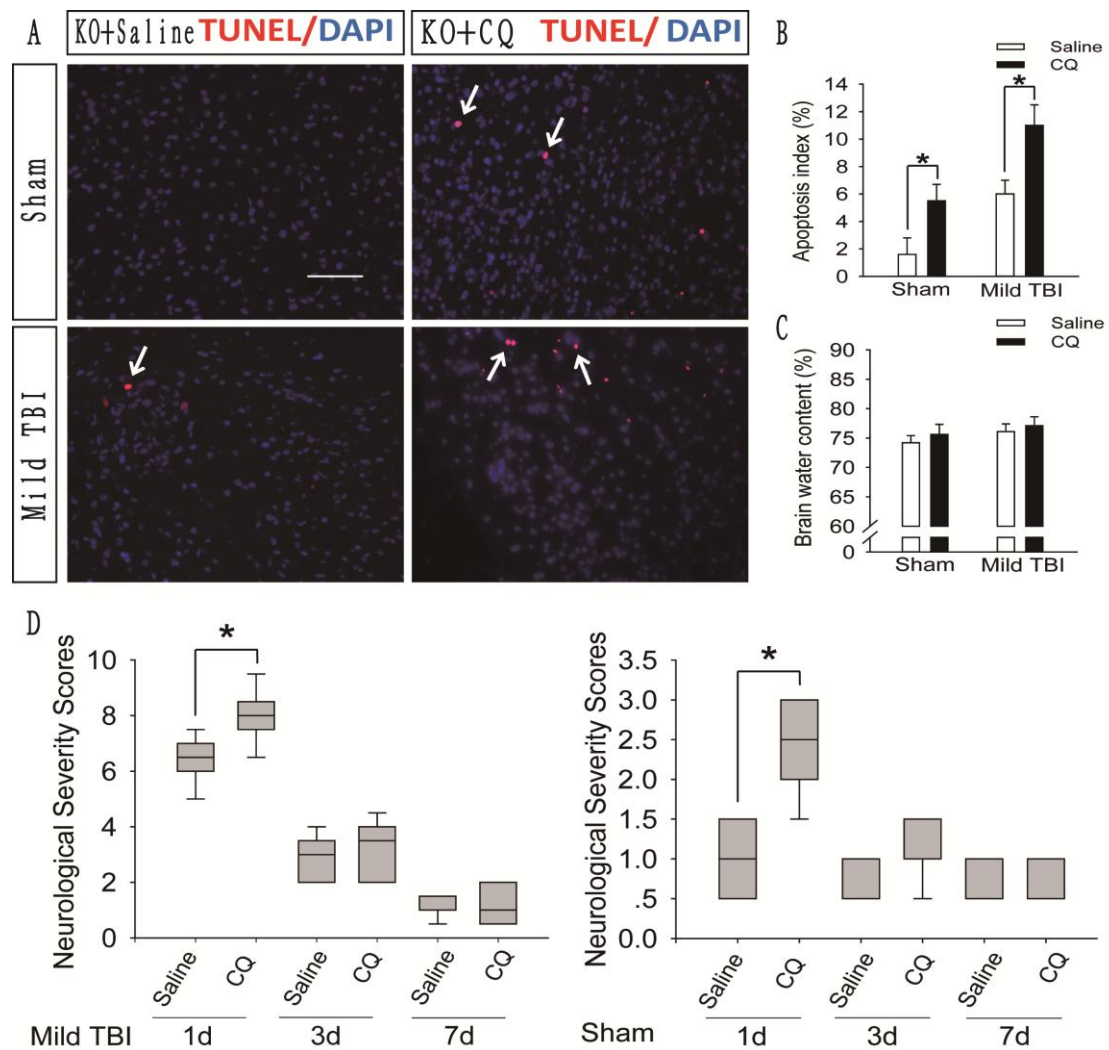

Supplement: Supplementary file 1 — Figure S1 [file 41419_2018_316_MOESM1_ESM.pdf]
