# Supplementary material for: Impaired autophagic flux is associated with the severity of trauma and the role of A2AR in brain cells after traumatic brain injury
Source: Cell Death Dis. 2018 Feb 14;9(2):252. doi: 10.1038/s41419-018-0316-4 (PMC5833790; doi:10.1038/s41419-018-0316-4)
Supplement: Supplementary file 2 — Figure S1 legend [file 41419_2018_316_MOESM2_ESM.doc]

**Figure S1.** CQ impairs autophagic flux in the injured cortex of A_2A_R KO mice after mild TBI and exacerbates the prognosis of brain injury. **(A)** Images of apoptotic cells in cortical brain sections obtained from A_2A_R KO mice subjected to sham surgery or mild TBI that were administered CQ or saline. The results of TUNEL staining performed 1 day after sham surgery or mild TBI are shown. Arrows indicate TUNEL-positive cells. Scale bar = 50 μm. **(B)** Quantification of the number of TUNEL-positive cells in the cortical brain sections shown in **(A)**. Data are presented as means ± SEM, n = 5-6, *P < 0.05 compared to the control group. **(C)** Brain water content of A_2A_R KO mice 1 day after mice were subjected to sham surgery or mild TBI and then administered CQ or saline. Data are presented as means ± SEM, n = 3. **(D)** Neurological severity scores in A_2A_R KO mice subjected to sham surgery or mild TBI and then administered CQ or saline. Scores were obtained at 1, 3 and 7 days after sham surgery or mild TBI, n = 7, *P < 0.05 compared to the control group at each time point.
